# SLFN11’s surveillance role in protein homeostasis

**DOI:** 10.18632/oncoscience.560

**Published:** 2022-07-25

**Authors:** Yasuhisa Murai, Ukhyun Jo, Yasuhiro Arakawa, Naoko Takebe, Yves Pommier

**Affiliations:** ^1^Department of Gastroenterology and Hematology, Hirosaki University Graduate School of Medicine, Hirosaki, Japan; ^2^Developmental Therapeutics Branch and Laboratory of Molecular Pharmacology, Center for Cancer Research, NCI, NIH, Bethesda, MD, USA

**Keywords:** Schlafen11, TAK-243, protein homeostasis, unfolded protein response, innate immunity

The endoplasmic reticulum (ER) is the organelle that produces functional proteins in eukaryotes. However, increased protein synthesis often causes protein misfolding, leading to ER stress and reciprocal activation of the unfolded protein response (UPR). The ubiquitin-proteasome system (UPS) and ER stress-associated protein degradation (ERAD) pathways remove immature proteins.

**Figure 1:**
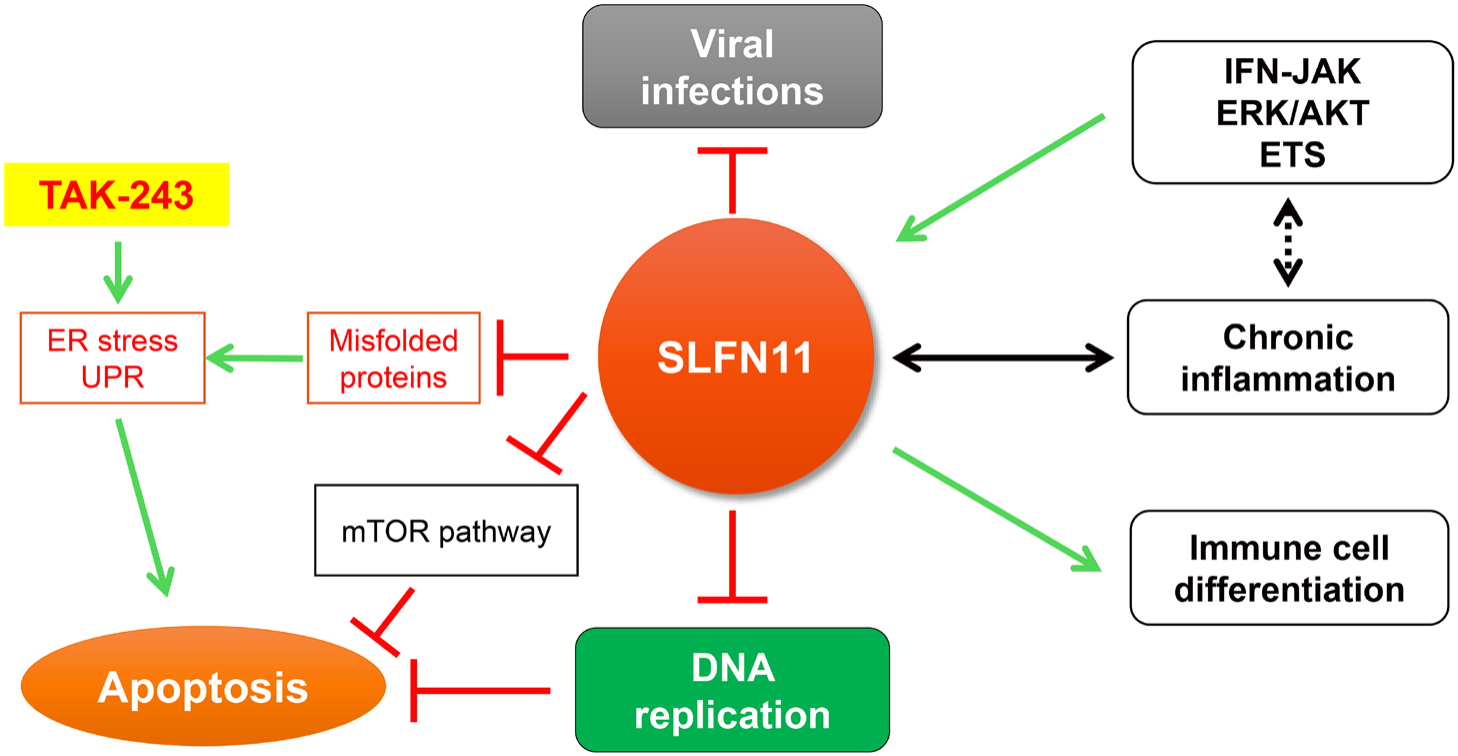
The multiple functions and interactions of SLFN11. Red bars indicate inhibition and green arrows show stimulation.

Recently, we demonstrated that *Schlafen11* (*SLFN11*) acts as a surveillance factor for protein homeostasis by alleviating the proteotoxic stress derived from protein synthesis and maturation [1]. *Schlafen* (“*to* *sleep*” in German) is the name of a family of genes encompassing *SLFN5,* *SLFN11, SLFN12*, *SLFN12L*, *SLFN13,* and *SLFN14* in human cells. Among the *SLFN* family, *SLFN11* has been identified as a critical determinant for the cytotoxicity of anticancer agents targeting DNA replication across multiple cancer types. SLFN11 is recruited to damaged replication forks under replication stress. It irreversibly inhibits replication by promoting the destabilization of Cdc45-Mcm2-7-GINS (CMG) helicase complex, degrading the Chromatin Licensing and DNA Replication Factor 1 (CDT1), remodeling chromatin, and inducing immediate early genes [2, 3]. Its lack of expression in ~50% of cancer cells leads to chemoresistance. SLFN11 also plays a pivotal role inhibiting viral infection and tumorigenesis [4, 5] (Figure 1).

By screening the NCATS drug library, containing 1978 compounds, we recently reported that TAK-243 (MLN7243), a first-in-class inhibitor of the ubiquitin-activating enzyme UBA1 (also known as UBE1) preferentially suppresses cell proliferation of *SLFN11*-deficient cancer cells [1]. TAK-243 binds free ubiquitin to form irreversible ubiquitin adducts and induces ER and proteotoxic stress [6], thereby leading to cancer cell death. We also found that cancer cells that do not express SLFN11 exhibit increased global protein ubiquitylation, ER stress and UPR compared to *SLFN11*-proficient cells. The increased susceptibility of *SLFN11*-deficient cells to TAK-243 was associated with an enhanced activation of the UPR transducers PERK, phosphorylated eIF2α, phosphorylated IRE1 and ATF6.

Given that phase 1 clinical trials with TAK-243 are underway in patients with advanced solid tumors and blood cancers (NCT02045095 and NCT03816319), our results imply that the expression status of SLFN11 might be utilized to predict therapeutic benefit of TAK-243 in cancer treatment. Regarding the mechanism of action, we identified that TAK-243-induced proteotoxic stress inhibits DNA replication by promoting Claspin-dependent CHK1 phosphorylation independently from ATR, RPA, and γ-H2AX activation. We also found interactions between SLFN11 and the protein synthesis machinery, including translation initiation factors (EIF3A, EIF3B, EIF3D, EIF3E, EIF3F, EIF3H, EIF3L, EIF3M, EIF4B, and EIF4G1) and protein folding related molecules (TCP1, CCT2, CCT3, CCT4, CCT5, CCT6A, CCT7, and CCT8). Taken together, our findings suggest that SLFN11 plays a role in protein homeostasis and that lack of SLFN11 expression makes cells vulnerable to anticancer drugs inducing ER and proteotoxic stress (Figure 1).

A detailed immunohistochemistry and RNA expression study recently showed that SLFN11 is expressed in normal human brain and immune cells, not only cancer cells [7, 8]. Uncontrolled ER stress can be causative of multiple human diseases such as atherosclerosis, diabetes, Alzheimer’s, and Parkinson’s diseases [9]. Given that SLFN11 is involved in protein homeostasis through the regulation of cellular protein-ubiquitin adducts, SLFN11 malfunctions may be associated with human diseases beyond cancer.

Furthermore, alteration of SLFN11 could be related to immune deficiency via regulation of immune response and inflammation (Figure 1). We recently reported that SLFN11 expression is regulated by the IFN-JAK pathway and its downstream MAPK(AKT/ERK)-ETS pathway [10]. SLFN11 expression is known to change during the differentiation of B-cell-derived cancers [8] and is associated with chronic intestinal mucosal inflammation and excessive apoptosis in organoid models and patient samples of ulcerative colitis [11]. These observations suggest that SLFN11 works as a co-regulator of immune cells and as an indicator of chronic inflammation. Therefore, further studies are warranted to investigate the role of SLFN11 expression in human autoimmune and inflammatory diseases and as a predictor biomarker of response for patients treated with TAK-243.
